# Track distance runners exhibit bilateral differences in the plantar fascia stiffness

**DOI:** 10.1038/s41598-021-88883-4

**Published:** 2021-04-29

**Authors:** Hiroto Shiotani, Ryo Yamashita, Tomohiro Mizokuchi, Natsuki Sado, Munekazu Naito, Yasuo Kawakami

**Affiliations:** 1 Faculty of Sport Sciences, Waseda University, Saitama, Japan; 2 Human Performance Laboratory, Comprehensive Research Organization, Waseda University, Tokyo, Japan; 3 School of Sport Sciences, Waseda University, Saitama, Japan; 4 Faculty of Health and Sport Sciences, University of Tsukuba, Ibaraki, Japan; 5 Department of Anatomy, Aichi Medical University, Aichi, Japan

**Keywords:** Musculoskeletal system, Ultrasonography

## Abstract

Human steady-state locomotion modes are symmetrical, leading to symmetric mechanical function of human feet in general; however, track distance running in a counterclockwise direction exposes the runner’s feet to asymmetrical stress. This may induce asymmetrical adaptation in the runners’ foot arch functions, but this has not been experimentally tested. Here, we show that the plantar fascia (PF), a primary structure of the foot arch elasticity, is stiffer for the left than the right foot as a characteristic of runners, via a cross-sectional study on 10 track distance runners and 10 untrained individuals. Shear wave velocity (index of tissue stiffness: SWV) and thickness of PF and foot dimensions were compared between sides and groups. Runners showed higher PF SWV in their left (9.4 ± 1.0 m/s) than right (8.9 ± 0.9 m/s) feet, whereas untrained individuals showed no bilateral differences (8.5 ± 1.5 m/s and 8.6 ± 1.7 m/s, respectively). Additionally, runners showed higher left to right (L/R) ratio of PF SWV than untrained men (105.1% and 97.7%, respectively). PF thickness and foot dimensions were not significantly different between sides or groups. These results demonstrate stiffer PF in the left feet of runners, which may reflect adaptation to their running-specific training that involves asymmetrical mechanical loading.

## Introduction

During human locomotion, the medial longitudinal arch of the foot is lowered while being stretched out in response to weight-bearing, and then recoils as the load is removed. Such a spring-like property of the foot arch helps to attenuate impact forces and store/release elastic strain energy leading to energy saving in running^1,2^. Previous studies indicate that the foot arch elasticity is attributed to the plantar fascia (PF)^1,3,4^. PF behaves viscoelastically under load^5,6^, and its resistive tension helps to prevent the lengthening and lowering of the foot arch. During each foot contact of running, PF is repetitively loaded with the tension reaching as high as 0.6–3.7 times bodyweight with its longitudinal strain up to 6%^7–10^. Such sizable stress concentrates around the proximal site of PF^11–13^, which may be associated with the heterogeneity of mechanical and morphological properties (e.g., stiffness and thickness) of PF^14–16^ as well as the occurrence of plantar fasciitis^17,18^.

The localized stiffness of PF can be quantitatively assessed as the shear wave velocity (SWV) in vivo^14–16^. PF has higher SWV (i.e., stiffer) at the proximal site than middle and distal sites^14,16^. Additionally, long-distance running induced a transient decrease of SWV at the proximal site of PF while long-distance runners showing smaller changes in SWV than untrained individuals^15^, suggesting that runners had built up a more resilient PF. These findings are evidence of PF adaptability to site-specific and chronic mechanical stress, which can be reflected in its stiffness and morphology.

Human steady-state locomotion modes are symmetrical, leading to symmetric mechanical function of human feet in general; however, track distance running is performed always in a counterclockwise direction, i.e., the left leg being inside during curve running. In this phase, runners are required to generate greater forces with their left legs^19,20^ to exert centripetal force^21^. This is associated with the greater load on the left foot, resulting in the lowering of the left foot arch, and thus leading to an increase of mechanical stress to PF. Therefore, runners’ feet can be exposed to asymmetrical stress during running. This may induce asymmetrical adaptation in runners’ PF stiffness and morphology. Although PF SWV and thickness, and the foot dimensions were comparable between left and right sides in a healthy and untrained population^14^, this may not be true for track distance runners. If the bilateral differences in runner’s feet can be confirmed, this provides an indication of a threshold of mechanical stress that causes adaptation of PF and foot arch functions. A profound understanding of PF adaptability is essential for improvements in their performance as well as prevention of plantar fasciitis.

Therefore, the purpose of this study was to investigate the bilateral differences in mechanical and morphological properties of PF and foot dimensions in track distance runners, as contrasted to untrained individuals. We hypothesized that track distance runners have bilateral differences in PF SWV, thickness, and the foot arch height, and that runners show sizable differences in the left to right (L/R) ratios of measured variables as compared to untrained individuals.

## Results

**Figure 1:**
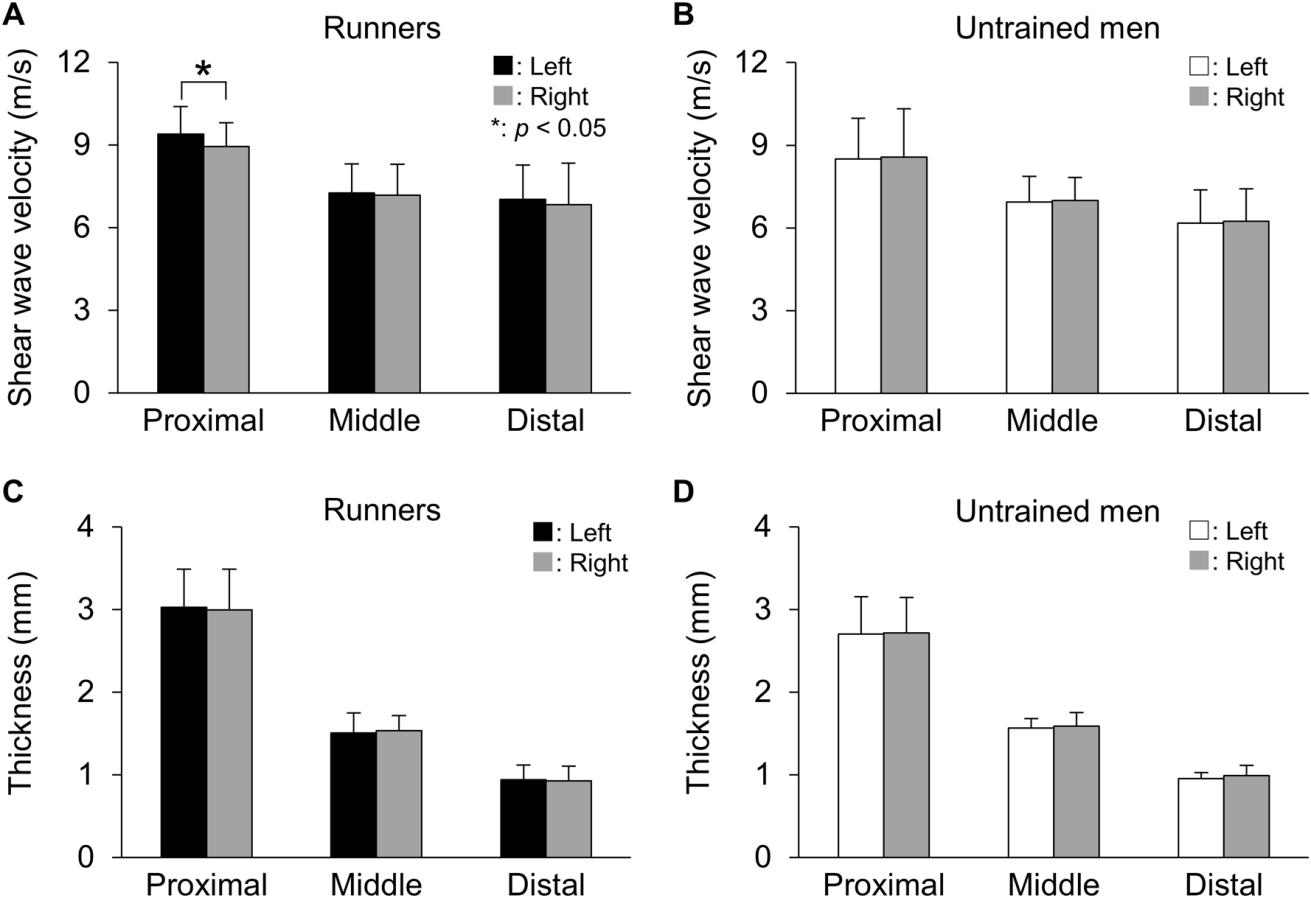
Bilateral differences in SWV and thickness of runners and untrained men. Data are shown as means ± SD.

In runners, SWV at the proximal site was significantly higher in left (9.4 ± 1.0 m/s) than right foot (8.9 ± 0.9 m/s) (*p* = 0.021, *d* = 0.813), but not at the middle (*p* = 0.782, *d* = 0.073) or distal sites (*p* = 0.554, *d* = 0.138) (Fig. 1). Even in a lefty runner (n = 1), PF SWV at the proximal site was higher for his left (10.0 m/s) than right foot (9.1 m/s). PF SWV at the proximal site was also higher for the left than the right feet both in rearfoot strike (n = 7, left: 9.0 ± 1.0 m/s and right: 8.6 ± 0.8 m/s, respectively) and forefoot strike runners (n = 3, left: 10.2 ± 0.3 m/s and right: 9.6 ± 0.5 m/s, respectively). In untrained men, SWV at each measurement site was not significantly different between left and right feet (*p* ≥ 0.222, *d* ≤ 0.264). PF thickness at each measurement site was not significantly different between left and right feet in runners (*p* ≥ 0.327, *d* ≤ 0.141) or untrained men (*p* ≥ 0.411, *d* ≤ 0.305) (Fig. 1). Foot dimensions were not significantly different between left and right feet in either of runners or untrained men (Table 1).

**Table 1:** Bilateral differences in foot dimensions of runners and untrained men.

	Runners (*n* = 10)			Untrained men (*n* = 10)		
	Left	Right	*p* value Cohen’s *d*	Left	Right	*p* value Cohen’s *d*
Foot length (mm)	245.3 ± 7.3	245.9 ± 8.6	*p* = 0.520	247.4 ± 8.7	248.2 ± 8.1	*p* = 0.445
			*d* = 0.075			*d* = 0.107
Dorsal height (mm)	61.1 ± 4.4	60.8 ± 4.2	*p* = 0.760	60.5 ± 5.5	61.1 ± 4.0	*p* = 0.671
			*d* = 0.070			*d* = 0.125
Navicular height (mm)	43.3 ± 6.2	41.9 ± 6.8	*p* = 0.080	41.3 ± 4.8	40.9 ± 5.4	*p* = 0.507
			*d* = 0.215			*d* = 0.078
Arch height ratio (%)	17.7 ± 2.7	17.1 ± 3.0	*p* = 0.064	16.7 ± 1.8	16.4 ± 1.9	*p* = 0.400
			*d* = 0.210			*d* = 0.162

Data are shown as means ± SD.

The L/R ratio of SWV at the proximal site was significantly higher in runners than untrained men (*p* = 0.027, *d* = 1.076), but not at the middle (*p* = 0.815, *d* = 0.107) or distal sites (*p* = 0.421, *d* = 0.369). The L/R ratios of thickness at any of the measurement sites or foot dimensions were not significantly different between groups (Table 2).

**Table 2:** Left/right ratios (%) of individual parameters in runners and untrained men.

Variable	Runners	Untrained men	*p* value Cohen’s *d*
*Shear wave velocity*			
Proximal	**105.1 ± 6.2**	**97.7 ± 7.6**	***p = 0.027***
			***d = 1.076***
Middle	101.8 ± 12.9	100.2 ± 17.2	*p* = 0.815
			*d* = 0.107
Distal	104.6 ± 13.7	99.7 ± 12.8	*p* = 0.421
			*d* = 0.360
*Thickness*			
Proximal	101.3 ± 6.4	99.6 ± 8.0	*p* = 0.610
			*d* = 0.232
Middle	97.8 ± 5.9	98.8 ± 8.7	*p* = 0.756
			*d* = 0.141
Distal	102.3 ± 14.1	97.6 ± 11.7	*p* = 0.429
			*d* = 0.362
Foot length	99.8 ± 1.1	99.7 ± 1.3	*p* = 0.843
			*d* = 0.083
Dorsal height	100.5 ± 3.9	99.1 ± 6.3	*p* = 0.583
			*d* = 0.267
Navicular height	103.9 ± 5.8	101.2 ± 4.4	*p* = 0.270
			*d* = 0.525
Arch height ratio	104.1 ± 6.0	101.6 ± 5.1	*p* = 0.329
			*d* = 0.449

Data are shown as means ± SD.

Bold fonts indicate significant difference between runners and untrained men (*p* < 0.05) with a “large” effect size (*d* ≥ 0.8).

Age, body height, body mass, BMI, and fractions of leg dominance and foot strike pattern were not significantly different between runners and untrained men (Table 3). All participants were healthy and free from injury of the lower extremity in the past 12 months and had no present or past history of plantar fasciitis. The runners had kept habitual running of at least 10 km/week for the past year, mainly on a running track, and their running experiences ranged between 9 and 16 years. Their personal best time of 5000 m ranged from 14′ 15 to 15′ 30. The untrained participants were either sedentary or lightly active, and none of them had been involved in any structured training program or continuous sports participation at least 12 months before the measurements. All participants used conventional running shoes rather than minimalist, high cushion, or high motion control shoes.

**Table 3:** Physical characteristics of participants.

Variable	Runners	Untrained men	*p* value
*n*	10	10	–
Age (years)	22.0 ± 0.7	22.5 ± 1.4	0.309
Body height (m)	1.68 ± 0.04	1.70 ± 0.05	0.392
Body mass (kg)	55.5 ± 4.2	58.4 ± 5.6	0.062
BMI (kg/m^2^)	19.6 ± 1.2	20.3 ± 1.7	0.113
Dominant leg (Lefty:Righty)	1:9	1:9	1.000
*Foot strike pattern (RFS:FFS)*	*7:3*	*10:0*	*0.060*
Running experience (years)	11.0 ± 2.2	–	–
Running distance (km/week)	43.7 ± 35.4	–	–

Data are shown as means ± SD.

*BMI* body mass index, *RFS* rear foot strikers, *FFS* forefoot strikers.

Age, body height, body mass, BMI, and fractions of leg dominance and foot strike pattern were not significantly different between runners and untrained men.

## Discussion

To the best of our knowledge, this is the first study to investigate bilateral differences in the mechanical and morphological properties of PF in track distance runners. The most striking finding of the present study was that track distance runners showed stiffer PF at the proximal site in their left than the right feet, unlike the untrained participants. A number of previous studies addressing running mechanics^22,23^ and mechanical and morphological properties of the musculotendinous and fascial tissues^15,24^ in runners have focused on unilateral leg by assuming bilateral symmetry. Our findings however suggest that asymmetry in runners is the major issue that needs to be carefully considered.

The greater load on the left foot during curve running^19,20^ can induce an increase of mechanical stress on PF. We previously revealed that running causes a decrease in PF SWV at its proximal site^15^, and this coincides with the simulation of stress distribution along PF^11–13^. The bilateral differences in PF stiffness at the proximal site in runners may reflect the adaptation to such stress accumulation in this region of the left foot during track running, regardless of the lateral dominance or foot strike patterns. The fact that PF stiffness in the right feet of runners was comparable to that of both feet in untrained individuals suggests a threshold of mechanical stress that causes adaptation of PF stiffness, which is side-specific for track runners. The finding that PF can be stiffened in response to a sufficient load is valuable for the general population toward injury prevention and rehabilitation as well as improvements in human locomotor performance.

Since the runners in this study had no history of plantar fasciitis, they might have been successful examples who had been optimally adapted to their running-specific training. However, plantar fasciitis is one of the most common injuries in long-distance runners, regardless of their performance levels^25,26^. This injury frequently occurs around the proximal site of PF^17,18^ where the mechanical stress is concentrated^11–13^. Thus, there is a clinical implication that the left foot of track runners can suffer from a higher incidence of this injury if their training adaptation does not work well. The bilateral imbalances in strength, morphology, and running mechanics are considered to be risk factors for injury of runners^27,28^. Interactions between these factors for the occurrence of plantar fasciitis are worth examining in future studies.

No bilateral differences in PF thickness of runners and untrained individuals are consistent with previous findings that PF thickness was not different between recreational runners and untrained individuals^15^, and that PF thickness was not influenced by physical activity^29^. These results, together with our findings, discard the possibility of PF adaptability in terms of its thickness for reducing mechanical stress induced by distance running. No bilateral difference in the foot arch dimensions suggests that the arches of both feet of runners can fulfill their imposed roles through different mechanical properties with comparable morphology.

We could not obtain the running mechanics and the foot arch deformation during running. This is one of the limitations of the present study. As our findings suggest a threshold of mechanical stress that causes adaptation of PF stiffness, quantifying the mechanical stress applied to bilateral feet during track running will lead to a better understanding of the nature of PF adaptability. Additionally, the runners who participated in the present study can be categorized as recreational level^30^. Runners of different performance levels (e.g., competitive and elite runners) show different running mechanics^31,32^ and fatigue responses^33,34^. Thus, competitive and elite runners have the possibility to exhibit different signs of adaptation in PF and foot morphology. Comparisons between runners in different performance levels should be incorporated in these future studies. Moreover, there is a variation in the training volume of runners (Table 3). As we previously reported that long-distance running induced transient decreases of PF SWV^15^, the training volume can be a potential factor that affects PF properties and their adaptation. In addition, the material and mechanical properties of the running surface (e.g., rubber, asphalt, or grass) as well as shoe sole have the possibility to affect the magnitude of stress to the foot^35–38^. Future studies addressing the chronic effects of training volume and environment on PF adaptation as well as foot arch functions are needed. Lastly, it can be assumed that sprinters, participating in the event of 200 and 400 m in particular, and long/high jumpers may also apply asymmetrical stress to their feet with a greater magnitude of stress compared to distance runners. Further investigation of bilateral differences in PF characteristics and foot dimensions in other events and sports athletes can be an option of the future theme in understanding PF adaptability.

In conclusion, this study showed bilateral differences in the mechanical but not in the morphological properties of PF and foot arch dimensions in track distance runners as compared to untrained individuals. PF SWV at the proximal site was higher in the left feet of track distance runners while their right feet showing comparable values to that of untrained individuals. These results demonstrate stiffer proximal PF in the left feet of runners, which may reflect adaptation to their running-specific training that involves asymmetrical mechanical loading.

## Methods

### Study design and participants

A cross-sectional study was conducted at Waseda University (Tokorozawa campus) in Japan from August to November 2017. This study was approved by the Human Research Ethics Committee of Waseda University (reference number: 2016-310) and was carried out in accordance with the Declaration of Helsinki. Written informed consent was obtained from all participants before data collection.

**Figure 2:**
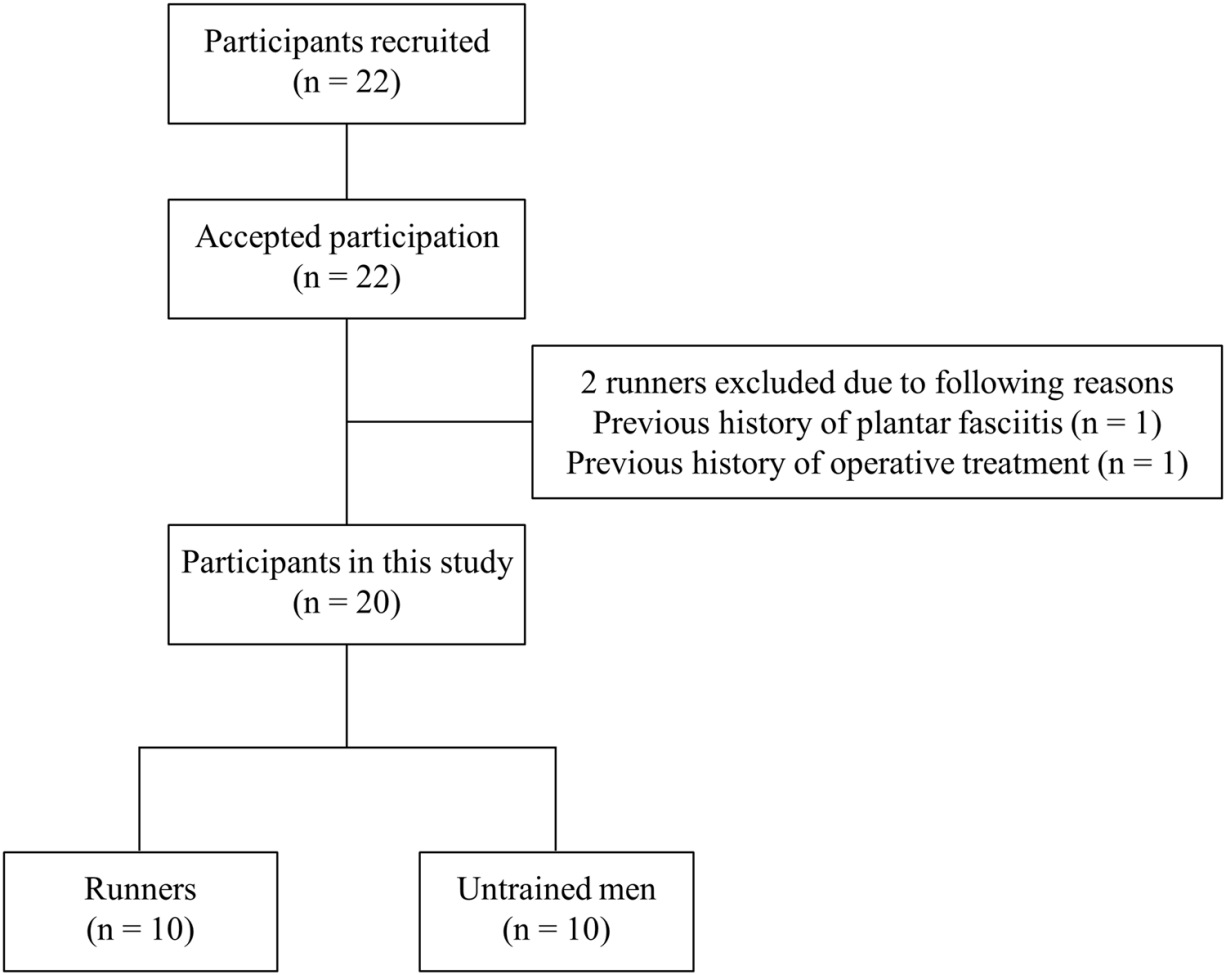
Flow diagram depicting participant selection. 12 runners were eligible for participation in this study. Two runners met the exclusion criteria of history of plantar fasciitis and operative treatment of the lower limb. Thus, 10 runners and 10 untrained men who matched the baseline physical characteristics with those of runners were included in this study.

The necessary sample size was calculated from our preliminary results (n = 6 in each group; total = 12). A priori power analysis (G*Power v3.1, Heinrich Heine-Universität Dusseldorf, Germany) with an assumed type 1 error of 0.05 and a statistical power of 0.80 was conducted to find significant differences in PF SWV between left and right feet of runners and between groups, respectively. The critical sample sizes were estimated to be at least 7 runners and 9 in each group (total = 18), respectively. Thus, 10 track distance male runners and 10 untrained men were recruited in this study (Table 1). Twelve runners were eligible for participation in this study. Of these, 2 runners met the exclusion criteria of history of plantar fasciitis and operative treatment of the lower limb (Fig. 2). Finally, 10 runners and 10 untrained men who matched the baseline physical characteristics with those of runners were successfully recruited in this study.

Before the main measurements, the profiles including age, body height, body mass, dominant leg, athletic experiences, exercise habits for the past year, foot strike pattern, history of injuries and operative treatment, and model of their running shoes were collected from all participants. The dominant leg was determined according to the participant’s favorite leg for kicking a ball. The foot strike pattern (rearfoot or forefoot strikers) of participants was visually confirmed on another occasion^15^. Additionally, the training environment (e.g., affiliation and running surfaces), personal best time of 5000 m, and running volume/week for the year were asked for runners. To avoid any confounding factors, we recruited participants attending the same university, and attempted to match the baseline physical characteristics of untrained participants with those of runners. Participants were not allowed to perform any strenuous exercises for at least 24 h before the measurement.

### Ultrasound measurements

The supersonic shear imaging (SSI) and B-mode ultrasonography techniques with an Aixplorer ultrasound scanner (version 6.4, Supersonic Imagine, Aix-en-Provence, France) and a linear array probe (SL 15-4, Supersonic Imagine, Aix-en-Provence, France) were used to measure the mechanical and morphological properties of PF. SSI is a valid and reliable technique to evaluate the stiffness of skeletal muscles, tendons, and fasciae in vivo^14,39–41^. In principle, SSI uses multiple push pulses to generate the shear waves propagating within the soft tissues and measures their velocity (i.e., SWV). Since SWV is related to Young’s modulus and shear modulus of the soft tissues, it can be used as an index of stiffness^42,43^.

**Figure 3:**
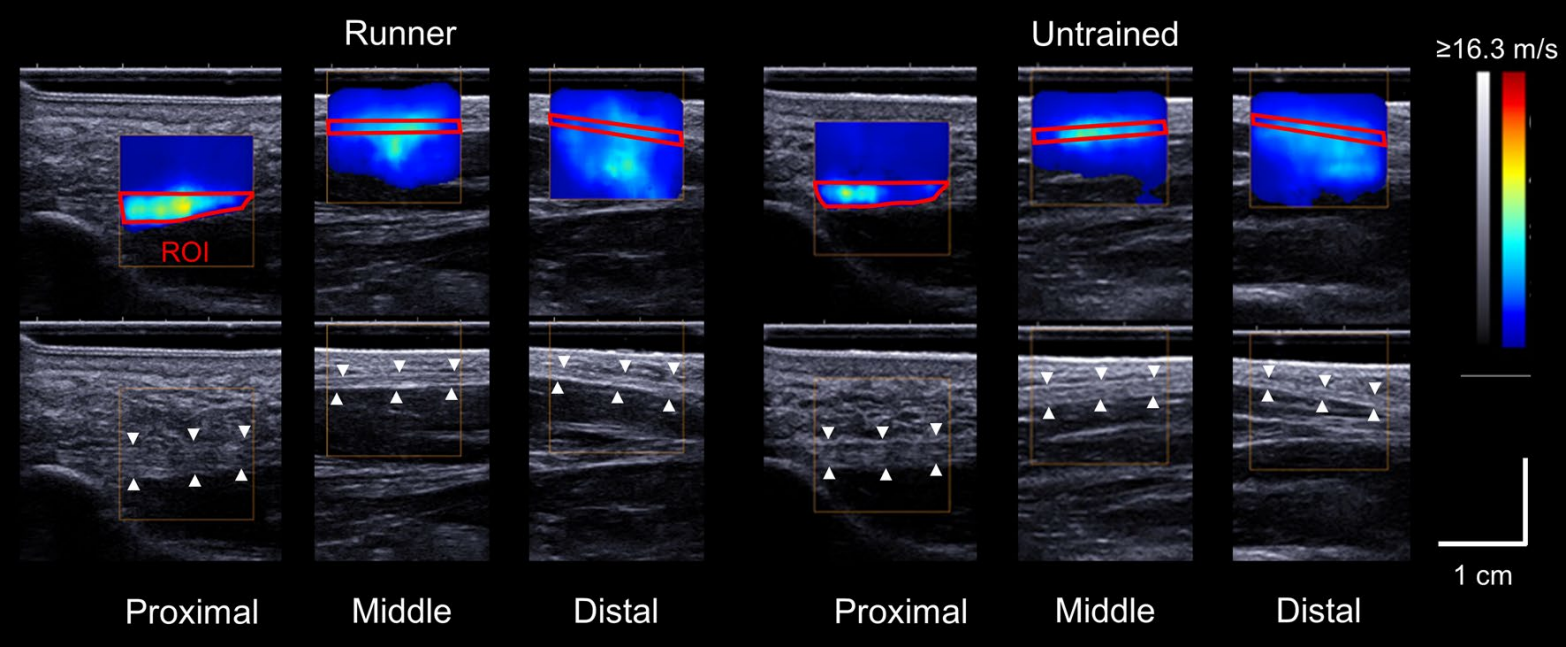
Representative shear wave and ultrasound B-mode images of the plantar fascia at the proximal, middle, and distal sites in a runner and an untrained participant. The region of interest (ROI) is bounded in red.

Details of SSI measurement and data processing were based on our previous published work^14,15^. During ultrasound measurements, participants were requested to rest in a supine position on the examination bed with their knee fully extended. Additionally, their ankle and toe digits were secured to a custom-made fixture at the neutral position. PF was scanned at three different sites along the longitudinal line between the medial calcaneal tubercle and the second toe. The locations of measurement sites were that at the proximal (in the proximity to the calcaneus), middle (the level of navicular tuberosity), and distal (proximity to the second metatarsal head) (Fig. 3). The longitudinal line of the foot and the locations of the transducer were marked on the skin surface using a waterproof marker. The scanning head of the probe was coated with transmission gel. An acoustic standoff pad (Gelpad for StatUS, Enraf–Nonius, Rotterdam, Netherland) was used to avoid applying excessive compression on the skin surface. Three images were obtained at each measurement site, and used for further analysis.

After data collection, SWV at each measurement site was measured as the mean value within the region of interest (ROI) which was manually traced over the fascial boundaries of PF using a measurement tool included in the Aixplorer software (i.e., Q-box Trace). PF thickness at each measurement site was measured the distance between the superficial and deep fascial boundaries was measured to determine thickness using a measurement tool (i.e., Distance). For SWV and thickness at each measurement site, three images were analyzed at each measurement site, then the three values were averaged to obtain the representative value.

### Measurements of the foot dimensions

A foot scanner (JMS-2100CU, Dream GP, Osaka, Japan) was used to obtain three-dimensional foot shape data. Details of measurement and data processing were based on our previous study using the same system^15^. Participants were requested to stand in a relaxed position with their feet approximately shoulder-width apart. The longitudinal axis of their feet, which is the line connecting between the most posterior point of the heel and the head of the second toe, aligned parallel with the guidelines drawn on the footplate in the foot scanner. A laser scanner moved around the foot in an oval trajectory, measuring the foot dimensions and the anatomical marker positions based on laser line triangulation. After the scanning, foot length, dorsal height, and navicular height were measured. The foot length was defined as the length projected on the longitudinal axis between the most posterior point of the heel and the head of the first or second toe, whichever was longer. The dorsal height was defined as the height of the highest point from the floor at 55% of the length of the foot from the heel. The navicular height was defined as the height of the most medial point of the navicular bone from the floor. Additionally, the arch height ratio was calculated as the navicular height normalized to the foot length.

### Statistical analysis

The normality of the data was assessed using a Shapiro–Wilk test. After the normality was confirmed, the difference in physical characteristics between groups were compared using an unpaired *t*-test. The fraction of dominant legs within each group was compared with a Pearson chi-squared test. Comparisons of measured variables between left and right feet in each group were performed using a paired *t*-test. The L/R ratios were calculated for the measured variables, and were compared using an unpaired *t*-test between groups. Cohen’s *d* was calculated as a measure of effect size. For the within-subject factor, it was corrected for dependence between mean values using the following equation: $d = {\text{M}}_{{{\text{diff}}}} /{\text{SD}}_{{{\text{pooled}}}} \sqrt {2\left( {1 - r} \right)}$, where ${\text{M}}_{{{\text{diff}}}}$ is mean difference between conditions, ${\text{SD}}_{{{\text{pooled}}}}$ is pooled SD, and $r$ is correlation between mean values^44^. Effect size is interpreted as trivial (*d* < 0.2), small (0.2 ≤ *d* < 0.5), medium (0.5 ≤ *d* < 0.8) and large effect (*d* ≥ 0.8)^45^. Statistical significance was set at α = 0.05. Statistical analysis was performed using SPSS software (SPSS Statistics 25, IBM, Armonk, USA).
